# Cancer Center Staff Satisfaction: Descriptive Results of a Canadian Study

**DOI:** 10.3390/curroncol30110717

**Published:** 2023-11-11

**Authors:** Rajiv Samant, Ege Babadagli, Selena Laprade, Gordon Emil Locke, Yuxin Zhang, Angela McNeil, Julie Renaud, Elisabeth Cisa-Paré, Jessica Chan, Jiheon Song, Joanne Meng

**Affiliations:** 1 The Ottawa Hospital Cancer Center, Ottawa, ON K1H 8L6, Canada; rsamant@toh.ca (R.S.); selelaprade@toh.ca (S.L.); golocke@toh.ca (G.E.L.); anmcneil@toh.ca (A.M.); jurenaud@toh.ca (J.R.); jmeng@toh.ca (J.M.); 2 Department of Radiology, Radiation Oncology and Medical Physics, University of Ottawa, Ottawa, ON K1N 6N5, Canada; 3 The Ottawa Hospital Research Institute, Ottawa, ON K1Y 4E9, Canada; yuxzhang@ohri.ca; 4 Northern Ontario School, Medicine University, Sudbury, ON P3E 2C6, Canada; ecisapare@nosm.ca; 5 Division of Radiation Oncology, University of British Columbia, Vancouver, BC V6T 1Z4, Canada; jessica.chan@bccancer.bc.ca; 6 BC Cancer, Vancouver, BC V5Z 4E6, Canada; 7 Department of Radiation Oncology, University of Toronto, Toronto, ON M5S 1A1, Canada; jiheon.song@sunnybrook.ca

**Keywords:** workplace, satisfaction, stress, burnout, oncology, cancer center, healthcare, staff

## Abstract

Caring for cancer patients is generally considered very rewarding work, but it can also be stressful and demanding. Therefore, it is important for oncology healthcare professionals to feel satisfied with their work environment in order to provide the best care possible. An ethics-approved 61-item staff satisfaction survey was developed in-house to gain insights regarding workplace satisfaction among all staff at The Ottawa Hospital Cancer Center. Descriptive statistics were used to analyze the responses. A total of 478 individuals completed the online survey, with 75.1% women, 23.2% men, and 1.7% preferring not to say. This represented the vast majority (>75%) of cancer center staff. The approximate breakdown according to healthcare professional type was as follows: 21% nurses, 20% radiation therapists, 18% physicians, 13% clerical staff, and 28% other types of staff. Almost all (97.4%) generally enjoyed their work, with 60% stating “very much” and 37.4% stating “a little bit”, and 93.3% found working with cancer patients rewarding. The overall satisfaction level at work was high, with 30.1% reporting “very satisfied” and 54.2% “somewhat satisfied”. However, in terms of their work being stressful, 18.6% stated it was “very much” and 62.1% “a little bit”. Also, in terms of their workload, 61.3% stated it was “very busy” and 10% stated it was “excessively busy”. The most enjoyable aspects of work were listed as interactions with colleagues, interactions with patients, and learning new things. The least enjoyable aspects of work were excessive workload, a perceived unsupportive work environment, and technology problems. Levels of satisfaction and stress at work varied according to role at the cancer center. Most cancer center staff seem to enjoy their work and find it rewarding. However, the work environment can be challenging and stressful. Areas for improvement include managing workloads, ensuring staff feel supported, and improving the user-friendliness of technology.

## 1. Introduction

The incidence of cancer is rising rapidly and consequently having a significant impact on healthcare systems around the world [1,2,3,4]. This poses a large burden in terms of both morbidity and mortality related to cancer, along with the challenges of keeping up with healthcare costs. The goal of oncology healthcare institutions has traditionally been to lessen this burden and ensure that high-quality care is offered and available to all patients. However, there is generally good consensus that it is becoming more difficult to achieve this, even in resource-rich countries.

It is also known that job dissatisfaction and work-related stress are on the rise and are becoming quite challenging for employers [5]. There are many causes for this, including excessive workload and work–life balance, but “unfair treatment at work” is usually considered the biggest component [5,6]. These can lead to a variety of negative physical, psychological, and emotional consequences [7]. Such problems are paralleled in the healthcare sector and can impact the sustainability of healthcare institutions [8]. In turn, there is a risk of a variety of downstream negative effects on healthcare providers and the patients they are looking after [9].

Additionally, it is increasingly being recognized that the wellbeing of healthcare providers impacts the care that patients receive, and this is also true in the oncology setting [10,11]. Even though working as an oncology healthcare professional can be incredibly gratifying, with both personal and professional rewards, there are also many challenges associated with caring for cancer patients. Multiple studies have confirmed the high levels of stress and burnout among oncology staff [12,13,14,15]. They indicate that there are a variety of issues that influence stress, burnout, and job satisfaction. These include individual and organizational factors, and they all need to be addressed.

The situation has been exacerbated over the last decade, especially since the COVID-19 pandemic, and it seems as though healthcare systems are in crisis [16]. These problems have been reported in oncology as well as in a variety of areas in medicine, and these include palliative care, emergency departments, intensive care units, and pediatrics [17,18,19,20,21]. Healthcare organizations are searching for ways to deal with these issues, and research is being conducted to find effective solutions [22,23]. However, there are no simple quick fixes, and a complicated, multi-pronged approach is necessary [24,25]. Also, interventions need to be targeted to individual staff needs in the workplace environment [26,27]. Therefore, assessing the needs of healthcare professionals is key to finding adequate solutions for the problems related to stress and burnout in the workplace.

Hence, an important aspect of improving cancer care needs to be ensuring that the most important component of healthcare systems, that is, the oncology staff, are sufficiently trained and assisted to do their work in a supportive and sustainable work environment [28,29,30]. Each cancer organization is unique, requiring individualized attention and approaches to improve staff wellbeing. The best way to start improving healthcare processes for oncology staff is to first assess and address the specific needs and concerns that exist.

At the Ottawa Hospital Cancer Center, the challenges of working in the oncology setting are well known, and these have been exacerbated since the worldwide COVID-19 pandemic began in 2020 [31,32]. Therefore, it has become a priority for cancer centers to address the needs of staff, and there has been keen interest in determining how best to support them. The published research shows both common issues of concern as well as differences, again highlighting that each organization must carefully assess its own unique circumstances [28,30,33,34].

An ad hoc group within the cancer center, consisting of frontline healthcare workers, managers, and administrators, decided to take formalized concrete steps to assess the situation within the facility. The goal was to see how staff viewed their work lives and to look for ways to improve working conditions based on their responses. It was also determined that it would be important to evaluate all staff, not just physicians and nurses who have been studied the most in the published literature [23,35], since it is crucial for the entire team to work effectively together to provide the high-quality care that is desired for patients. It was hypothesized that different groups of staff would not all have the same issues or concerns, and strategies supporting each group would be unique to their needs. This multidisciplinary team decided to create a staff survey tool designed to assess various aspects of the work environment, including job satisfaction, workload, stress, burnout, and patient interactions.

On review of the existing literature, there do not seem to be any published series documenting surveys of entire cancer center healthcare staff in the outpatient setting, with most published series focusing on just physicians, nurses, or radiation therapists being surveyed. As such, this is one of the first staff survey studies that targets all types of healthcare providers working in a tertiary care outpatient cancer program.

## 2. Materials and Methods

An ethics-approved 61-item staff satisfaction survey was developed by a multidisciplinary team of HCPs working within the radiation medicine program at The Ottawa Hospital Cancer Center. The survey was a cross-sectional study targeting healthcare staff affiliated with the outpatient cancer program, notably the only dedicated cancer program and referral center in the Ottawa-Carleton region, which serves a population of 1.3 million people. Healthcare staff in the inpatient programs were not involved. Although the exact number of healthcare staff working at the outpatient cancer program fluctuates, it was approximately between 600 and 700 individuals at the time of the study. Targeted physicians included radiation oncologists, medical oncologists, hematologists, surgeons, palliative care specialists, and general practitioners in oncology, including residents and fellow trainees. 

The questionnaire was reviewed by physicians, residents, nurses, radiation therapists, and administrators and was specifically designed to be quite inclusive of all cancer center employees. It included demographic information and evaluated a variety of domains, including staff satisfaction, stress, burnout, coping strategies, and patient interactions. The Likert scale was utilized for the majority of questions in the survey, while other questions required “yes” or “no” responses, and some questions required free-form text input. Validated instruments to measure satisfaction, stress, or burnout were not utilized, instead, in-house questions to assess satisfaction, stress, burnout, and other factors influencing the workplace experience were designed in-house. The reason for this was that the inclusion of validated instruments would have significantly increased the number of questions included in the survey and could have hampered completion rates.

The survey was electronic, anonymous, voluntary, and required approximately 15 min to complete. It was sent to staff at the cancer center via an email link with the use of a common emailing list. Respondents were allowed to complete the survey only once via email verification, and their responses were taken at face value. Respondents were allowed to provide their thoughts and opinions with no explicit assessment of the accuracy of their statements. A USD 10 gift card incentive was offered for the completion of the survey. An email invitation to complete the survey was sent out in November 2020, along with a maximum of 3 reminder emails at approximately weekly intervals. 

The survey responses were collated in an Excel spreadsheet, and descriptive statistics were utilized to generate the results. A series of preliminary analyses were conducted to identify relationships between the various variables and demographics, including age, gender, job category, and years of experience at the cancer center. Although the majority of the analysis conducted was based on a review of survey questions requiring selection of pre-defined responses based on the Likert scale, questions requiring free-form text input were also summarized to identify common themes. 

This study was meant to be descriptive and qualitative in nature; therefore, inferential statistics were not a focus of its analysis. Nonetheless, a few interesting correlations have been included when appropriate to highlight certain patterns among the study population. Correlations were determined using Pearson’s Chi-square test; a *p* < 0.05 threshold was used for statistical significance. 

This study was conducted in accordance with the Declaration of Helsinki and approved by the Research Ethics Board of The Ottawa Hospital on 20 May 2020 (20200084-01H).

## 3. Results

A total of 478 respondents completed the survey, with 95.4% stating they were primarily working at the cancer center. This represented over 75% of cancer center staff at the time. The breakdown according to gender was 71.5% women, 23.2% men, and 1.7% preferring not to say. The median age range was 41–50 years, and 76.8% were either married or in common-law relationships. Over half (64.3%) had worked at the center for at least 5 years, with 47.1% having worked for 10 or more years. The approximate breakdown according to healthcare professional type is shown in Table 1 and is as follows: 21% nurses, 20% radiation therapist, 18% physician, 13% clinical staff, and 28% other types of staff. The majority were associated with either the Radiation Medicine program (36.6%), the Outpatient Clinics (29.9%), or the Systemic Treatment Program (11.1%). The vast majority of respondents (93.3%) had some degree of patient interaction within their jobs, with 74.9% describing it as moderate or high.

**Figure 1:**
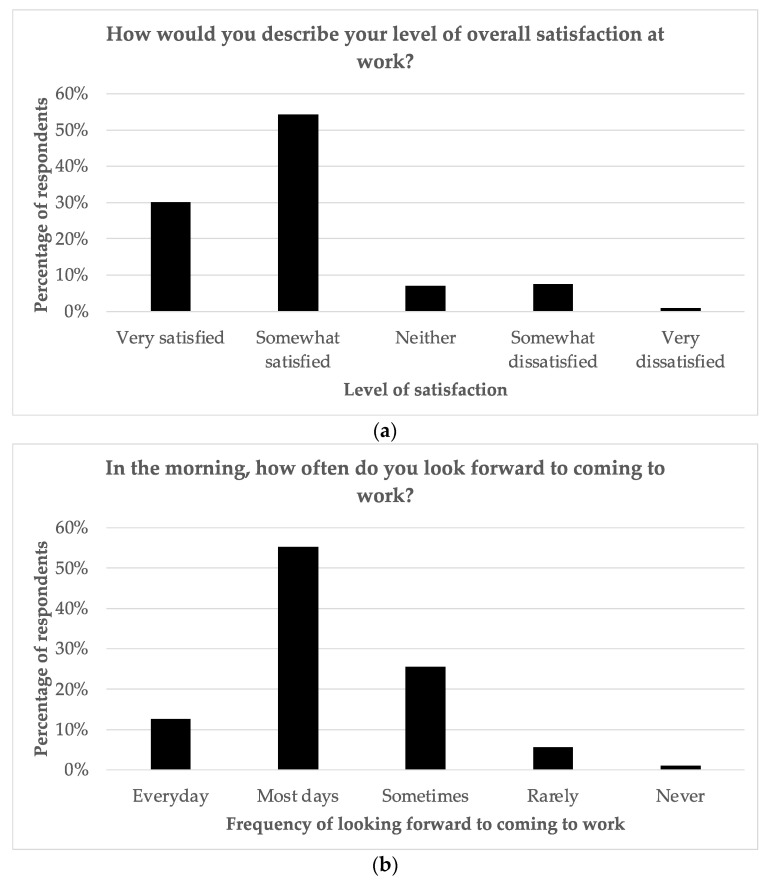

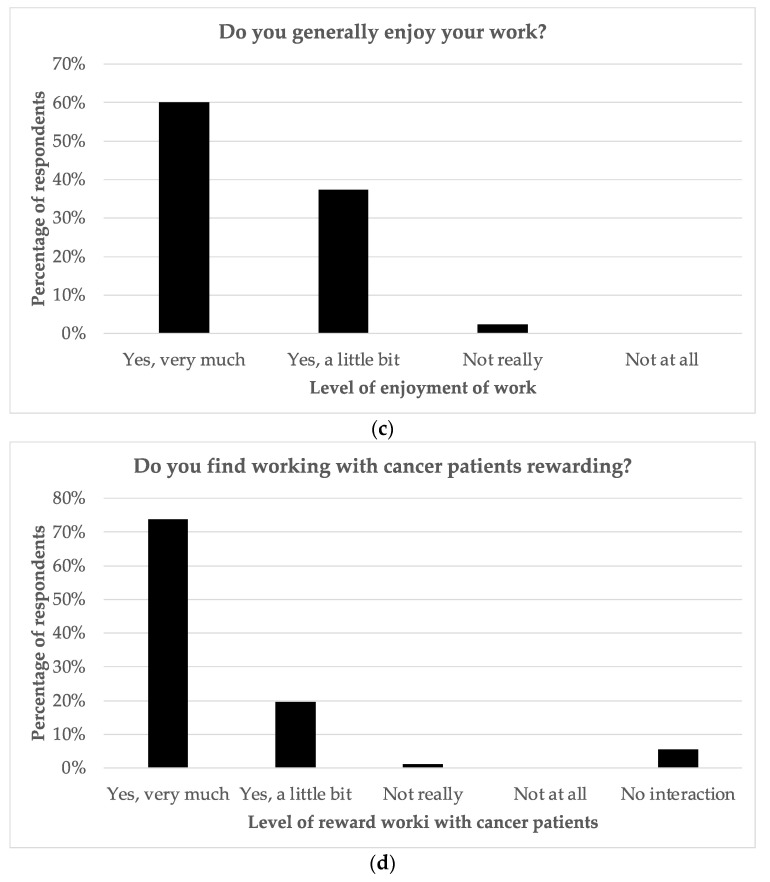
Survey responses regarding (**a**) level of satisfaction at work; (**b**) frequency of looking forward to coming to work; (**c**) level of enjoyment of work; (**d**) level of reward working with cancer patients.

Figure 1 indicates that almost all (97.4%) generally enjoyed their work, with 60% stating “very much” and 37.4% stating “a little bit “, and 93.3% finding working with cancer patients rewarding. The overall satisfaction level at work was high, with 30.1% reporting “very satisfied” and 54.2% reporting “somewhat satisfied “. Over two-thirds looked forward to coming to work, either “most days “(55.2%) or “every day” (12.6%). Managers reported the highest level of workplace satisfaction, whereas pharmacists reported the lowest levels. The level of satisfaction in general was correlated with age, with those aged over 60 years demonstrating higher levels of satisfaction (*p* = 0.038). Satisfaction was also correlated with the number of years worked, with those having worked 0–2 years having the highest levels of satisfaction and those having worked more than 20 years having the lowest levels of satisfaction (*p* = 0.002). Interestingly, the level of satisfaction was not correlated with workload. 

**Figure 2:**
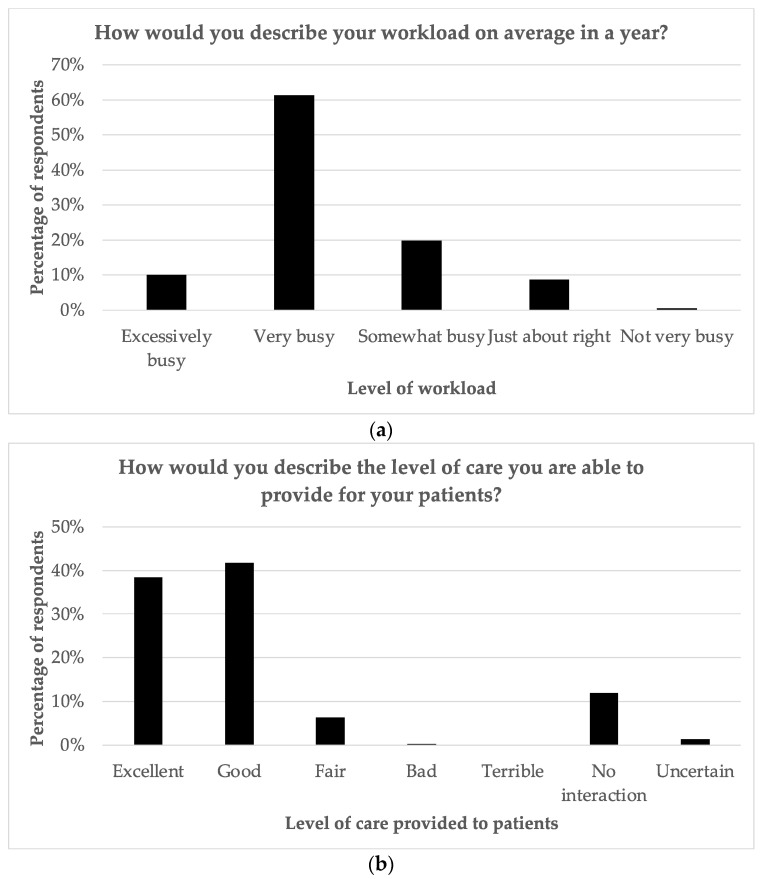
Survey responses regarding (**a**) average level of workload; (**b**) level of care provided to patients.

Figure 2 shows that approximately 80% of staff believe that they were able to provide either “Good “or “Excellent “care to patients. Also, in terms of their workload, 61.3% stated it was “very busy “and 10% stated it was “excessively busy “. Approximately half of the staff (50.2%) felt that they were able to spend enough time dealing with the specific needs of their cancer patients. The majority of staff felt that patients should be involved in decision-making with regard to care and also wanted to be involved. They also stated that giving patients hope was either “somewhat important” or “very important “. They rated the most important qualities of healthcare providers as knowledge, kindness, being a good communicator, being a good listener, and honesty, respectively (in descending order of importance). 

The most enjoyable aspects of work, shown in Table 2, were listed as interactions with colleagues, interactions with patients, learning new things, personal achievements, and acknowledgment from others. The least enjoyable aspects of work were technology problems, an unsupportive work environment, excessive workload, no future prospects, and the design of clinic/workspace areas. 

**Figure 3:**
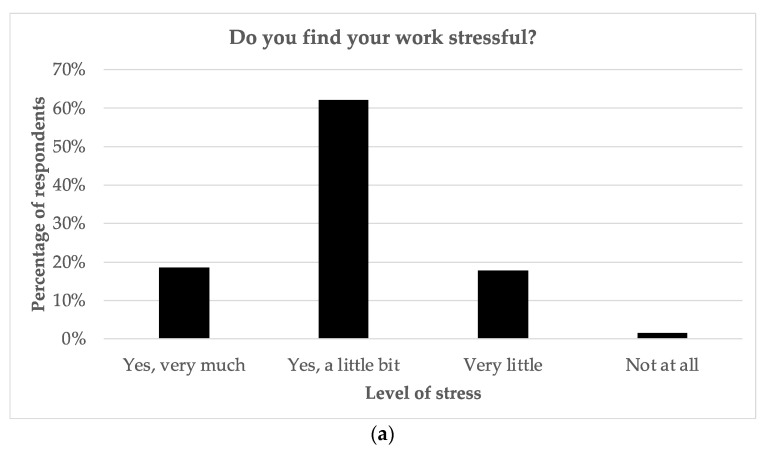

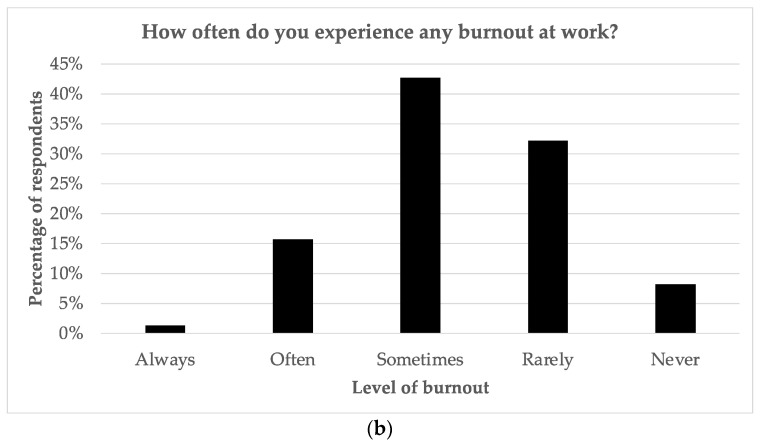
Survey responses regarding (**a**) levels of stress; (**b**) levels of perceived burnout.

As seen in Figure 3, most staff found their work stressful, with 18.6% stating “very much “and 62.1% stating “a little bit “. Resident and fellow physicians reported the highest levels of stress, while secretaries reported the lowest levels. Approximately one-quarter did regret their career choice at least sometimes, and 13.2% were considering changing careers at present. A total of 28.9% of respondents admitted to calling in sick because of work-related stress. Interestingly, while physicians were among those reporting the highest degree of stress, they were also among the least likely to call in sick for work related to stress. Physicians were also less likely to consider changing careers than most other groups. Overall, consideration of a career change was correlated with years worked, with those having worked more than 20 years more likely to have considered changing careers (*p* = 0.001). The most common strategies to cope with stress included taking breaks during the workday, exercise, mindfulness-type activities (breathing exercises, yoga, meditation, and prayer), family support, and interaction with colleagues. 

With regard to burnout (indicated in Figure 3), 16.9% of staff stated that they felt burned out either “often” or “always”, with 13.4% stating they were burned out at present and 25.7% answering “Maybe “. The majority (65.3%) admitted to having felt burned out in the past. Radiation therapists reported the highest perceived levels of burnout, whereas secretaries reported the lowest perceived levels. Burnout was correlated with lower satisfaction (*p* < 0.001), higher stress (*p* < 0.001), a higher workload (*p* < 0.001), and calling in sick due to stress at work more frequently (*p* < 0.001). Less than one quarter of staff (23.9%) believed there was enough support at work with regard to stress and burnout. 

Levels of satisfaction and stress at work varied according to the role the respondent played at the cancer center. Similarly, the most common likes and dislikes varied according to role. As highlighted in Table 3, technology problems were the most challenging for physicians, whereas the busy workload was most difficult for pharmacy staff and managers. Table 4 lists the most common suggestions for improving the workplace experience. 

## 4. Discussion

The results of this study are generally consistent with many published series, reporting significant levels of stress and perceived burnout among cancer center staff and other healthcare professionals [14,15,36,37,38]. This is even though most staff do enjoy their work and derive satisfaction from it. It was, however, interesting to note that approximately 60% enjoyed their work “very much”, yet only 30% stated they were “very satisfied” at work. This suggests that staff feel their work is valuable and gives them a sense of purpose, but clearly there are challenges within the work environment. This was seen among all groups of healthcare providers at the cancer center though burnout, stress, and job satisfaction varied among the respondent groups. 

The published data also suggest that burnout and stress negatively influence the quality of patient care that oncology staff can provide [39,40,41,42]. Interestingly, it was found that levels of stress were not correlated with the perceived quality of care provided by staff. The most significant issues at work depended on the roles of staff but included those commonly published elsewhere, such as computer/technology problems/challenges, an unsupportive work environment, and excessive workload [2,13,34]. Only half of the staff felt they were able to spend enough time with patients, and this is no doubt related to workload issues as well. Future career prospects and the design/functionality of their workspaces were also of concern to many staff. 

These findings highlight that major structural changes are needed to deal with the most pressing concerns of staff, and this has been reported by others [30,43,44,45,46,47]. Also, the needs of staff must be individualized. For example, technology problems were of most concern for physician respondents, whereas an unsupportive work environment was a bigger issue for nurses and radiation therapists, and workload was the major challenge for clerical and pharmacy staff. Clearly, the solutions are not simple and require a multifaceted approach. The literature suggests three general categories of interventions are often needed. These consist of individual, organizational and cultural measures [48]. Also, these can take time and resources to implement.

Some of the problems identified by staff are easier to address and do not require significant financial resources; these include increased teamwork, more effective communication, and the provision of directed positive feedback towards staff. Even such relatively minor improvements can potentially have a dramatic impact on the workplace environment. Modifications like these signal to staff that administration is concerned about their wellbeing and that they are making an effort to address the needs of staff. 

Other important changes, such as managing staff workloads, allowing for flexibility in schedules, dealing with technology problems, and financial compensation, are also very important and need to be dealt with. However, these changes require more resources and will likely take a longer time to address. Staff are likely aware of this, and will presumably remain patient if it is evident that some progress is being made. Many of the staff at the cancer center commented that they were appreciative that their opinions and views mattered through the questions that were asked in the survey. This suggests that merely requesting staff feedback can have a positive impact on workplace satisfaction and that administration is actively engaged in working on solutions. 

It is clear that a seismic cultural shift is required in the way cancer centers and other healthcare organizations view their roles and functions [13,14,28,49]. Although looking after the needs of patients is the raison d’etre for their existence, these institutions also need to view the needs of their staff as being of paramount importance. It also needs to be highlighted that some groups of staff are at greater risk than others, and therefore approaches need to be focused. Almost one-third of the respondents admitted to calling in sick for work-related stress. This should be of concern to employers. Perhaps, even more importantly, most staff do not believe there is enough support to deal with their concerns at work, and again, this is not new, as noted in the published literature [30,33,34,50]. 

Dealing with these issues is daunting, but not impossible. The good news is that most healthcare staff do enjoy their work, especially with regard to interactions with patients and colleagues, learning new things, and the acknowledgement they receive from others. There is a need to capitalize on these types of experiences while still addressing the significant challenges in the work environment. Clearly, increased resources will need to be devoted to improving the work environment, but some areas, such as providing staff opportunities to learn new things, acknowledgement for the valuable work they do, improving communication, and promoting more teamwork, are not expensive to deal with. However, this does require a mind-shift in the approach used to deal with workplace concerns, especially among supervisors, managers, and senior administration, to dealing with frontline healthcare staff. 

It has been established that stress and burnout in healthcare can lead to reduced quality of care provided for patients [40,51], so there is an urgent need to confront these issues, and the COVID-19 crisis seems to have vividly highlighted this. Making the necessary changes to restructure and reorganize the ways in which cancer care is provided cannot be delayed, and it is important to remember that staff are the most important components of the entire process. Therefore, the wellbeing of staff should really be a quality indicator for all healthcare organizations. 

This study does have some limitations, and these could limit the generalizability of the findings. Firstly, there could be a response bias, and it is uncertain whether stress and/or burnout among staff would influence their willingness to participate in the survey. The high participation rate (with approximately 75% completing the survey) suggests that the responses likely represent the full and accurate range of views of staff, though this is not absolutely certain. It was decided not to utilize standardized and validated tools to individually assess job satisfaction, stress, and burnout amongst staff, as this would have significantly increased the number of questions being asked. It was felt that self-perception of satisfaction, stress, and burnout levels would still be appropriate surrogates. The fact that these results and responses are entirely consistent with the published literature is therefore reassuring that this approach was reasonable. Also, the survey was conducted during the COVID-19 pandemic, and it is uncertain whether their responses would have been the same otherwise. However, though the survey tool was developed prior to the COVID-19 crisis, it was not possible to delay administration to a later date, as clearly the pandemic has lasted longer than most experts had originally predicted. Finally, some staff did question whether the survey was totally anonymous despite being given assurances, and this might have influenced respondent answers and comments. Clearly, more research is required to deal with this important problem that affects healthcare. The focus needs to be on finding adequate solutions and achieving commitment from healthcare institutions as well as buy-in from staff. It will also be important to regularly monitor the impact of workplace changes to determine if they are being effective.

## 5. Conclusions

This study indicates that the cancer center staff generally enjoy their work and derive satisfaction from it. However, levels of stress and burnout are concerning, and there is an urgent need to improve the workplace environment. Specific issues that need to be dealt with include managing excessive workloads, tackling technology challenges, and creating a highly supportive environment for staff where they feel valued.

## Figures and Tables

**Table 1:** Demographics of study participants.

Gender	Number of Participants	Percentage of Participants
Women	359	71.5%
Men	111	23.2%
Prefer not to say	8	1.7%
**Age**	**Number of Participants**	**Percentage of Participants**
30 or less	74	15.5%
31–40	130	27.2%
41–50	144	30.1%
51–60	104	21.8%
61+	19	4.0%
Prefer not to say	7	1.5%
**Marital Status**	**Number of Participants**	**Percentage of Participants**
Married	367	76.8%
Single	86	18.0%
Other	8	1.7%
Prefer not to say	17	3.6%
**Healthcare Role**	**Number of Participants**	**Percentage of Participants**
Physicians	89	18.6%
Nurses	102	21.3%
Radiation therapists	96	20.0%
Clerical	60	12.6%
Secretaries	21	4.4%
Clinical trials	25	5.2%
Managers	18	3.8%
Pharmacists	25	5.2%
Psychosocial oncology	14	2.9%
Physics	20	4.2%
Miscellaneous	8	1.7%
**Associated Program**	**Number of Participants**	**Percentage of Participants**
Radiation medicine program	175	36.6%
Outpatient clinics	143	29.9%
Systemic therapy unit	53	11.1%
Psychosocial oncology	18	3.8%
Supportive/palliative care	12	2.5%
Other	77	16.1%
**Number of Years Worked**	**Number of Participants**	**Percentage of Participants**
0–2	91	19.0%
2–5	80	16.7%
5–10	82	17.2%
10–20	136	28.5%
20–30	66	13.8%
30+	23	4.8%

**Table 2:** Enjoyable and unenjoyable aspects of work.

Enjoyable Aspects of Work	Frequency of Mention	Ranking
Interaction with colleagues	409	1
Learning new things	386	2
Interaction with patients	373	3
Personal achievements	225	4
Acknowledgement from others	201	5
Supportive work environment	181	6
Workload (busy schedule)	165	7
Adequate financial compensation	158	8
Academics (research, teaching)	134	9
Administration	49	10
**Unenjoyable Aspects of Work**	**Frequency of Mention**	**Ranking**
Technology problems	174	1
Unsupportive work environment	139	2
Workload (busy schedule)	125	3
No future prospects	108	4
Design and functionality of work area	107	5
Administrative paperwork	97	6
Lack of acknowledgement from others	92	7
Inadequate financial compensation	67	8
I cannot explain; everything is great	62	9
Same daily routine	46	10

**Table 3:** Most common dislikes about work according to role.

Role	Dislikes about Work
Physicians	Technology problems
Radiation therapists	Unsupportive work environment
Nurses	Unsupportive work environment, workload
Pharmacists	Workload
Physicists	No future prospects
Secretaries	Unsupportive work environment, workload
Clinical trials staff	Unsupportive work environment, workload
Managers	Workload
Clerical staff	Unsupportive work environment

**Table 4:** Most common coping strategies, reasons for considering career change, and suggestions for improving work experience.

Most Common Coping Strategies
Exercise/walking/spending time in nature Work breaks
Social/colleague support Meditation/mindfulness/yoga/prayer
Vacation/time off
Time-management strategies
Sleep
Music
Food
Animals
Work–life balance
**Reasons for Considering a Career Change**
High stress levels at work
Low pay
Excessive workload
Limited options for advancement
Feel undervalued
Feel unsupported at work
Lack of work flexibility
New career prospects/challenges
Healthcare cuts
**Suggestions for Improving Work Experience**
Workplace flexibility
Workload management/expectations
More support from management/feeling valued/respected
Fewer meetings
Better staff engagement
Decent staff rooms
More teamwork
Better communication
Increases in financial compensation

## Data Availability

The data presented in this study are not publicly available due to privacy reasons concerning study participants.
